# The ethics of using placebo in randomised controlled trials: a case study of a *Plasmodium vivax* antirelapse trial

**DOI:** 10.1186/s12910-018-0259-4

**Published:** 2018-03-06

**Authors:** Phaik Yeong Cheah, Norbert Steinkamp, Lorenz von Seidlein, Ric N. Price

**Affiliations:** 1 Mahidol Oxford Tropical Medicine Research Unit, Faculty of Tropical Medicine, Mahidol University, 420/6 Rajvithi Road, Bangkok, 10400 Thailand; 2 Centre for Tropical Medicine and Global Health, Nuffield Department of Clinical Medicine, University of Oxford, Oxford, UK; 3 The Ethox Centre, Nuffield Department of Population Health, University of Oxford, Oxford, UK; 4 Ethical Foundations of Social-Professional Practice, Catholic University of Applied Social Sciences, Berlin, Germany; 5 Global and Tropical Health Division, Menzies School of Health Research and Charles Darwin University, Darwin, NT Australia

**Keywords:** Ethics, Placebo, Vivax, Malaria, Randomised controlled trial

## Abstract

**Background:**

The use of placebos in randomised controlled trials is a subject of considerable ethical debate. In this paper we present a set of considerations to evaluate the ethics of placebo controlled trials that includes: social value of the study; need for a randomised controlled trial and placebo; standards of care; risks of harm due to administration of placebo and the harm benefit balance; clinical equipoise; and double standards. We illustrate the application of these considerations using a case study of a large ongoing multicentre, placebo-controlled, double-blinded, randomised trial to determine primaquine anti-relapse efficacy in vivax malaria.

**Main Body:**

There is an urgent need for primaquine anti-relapse studies in order to rationalise the management of a potentially fatal disease. An ethical justification for the use of the placebo arm is provided on the grounds that the actual current applied standard of care in most endemic places does not include primaquine. It has also been argued that there is clinical equipoise among the primaquine study arms and that the risk of harms of being in the placebo arm is the risk of having relapse, which is no more than not being included in the trial, and that there are no double standards.

**Conclusion:**

Based on our set of considerations, we conclude that a placebo arm is not only justified but imperative in this study. We propose that similar considerations should be prospectively applied to other placebo controlled trials and observational control arms where no treatment is offered.

## Background

For randomised controlled trials (RCTs) to yield valuable results, the choice of the control group is critical. Since the disease as well as the intervention effect measures of health outcome, researchers must compare the effects of an experimental intervention on participants assigned to the investigational arm (or arms) of a trial with the effects associated with a control arm. Randomisation is the preferred method for assigning participants to a treatment arm because it increases the likelihood that measured and unmeasured factors influencing the outcome are equally distributed between study arms. A control arm helps ensure that the results of the study reflect the effects of administered interventions and extraneous factors rather than the disease process itself [1].

The use of placebos as controls in randomised controlled trials is the subject of considerable ethical debate. The ethical dilemma is particularly apparent in resource-limited settings and infectious diseases where it hinges upon the issue of “double standards”; there is one moral standard governing research in the developed world and an inferior standard for research in developing countries. A series of placebo-controlled trials on mother-to-child transmission of Human Immunodeficiency Virus (HIV) in the late 1990s brought this to the fore [2]. These studies were conducted in Sub-Saharan Africa at a time when an effective, albeit expensive treatment to reduce this transmission was already available in the developed world. The studies caused public debate due to concerns that double standards of clinical care were being applied to individuals from the developed and developing world, in disregard of the right to equal access to optimal clinical care.

More than twenty years after the controversial HIV trials in Sub-Saharan Africa, researchers and research ethics committees still cannot agree on when placebos should or should not be used. This disagreement was illustrated recently by the debate over the design of Ebola trials in West Africa in which proponents of placebo argued that placebo or “standard of care” arms were permissible, and in fact imperative due to the unavailability of a proven treatment [3, 4]. It was argued that a placebo-controlled trial would produce the highest quality evidence in the most efficient manner, therefore maximizing the probability of saving future lives. However, opponents of placebo argued that standard care would offer little benefit as mortality was as high as 70%, the affected populations were frightened of the progress of the epidemic and it would be highly unlikely that a placebo arm would be acceptable. Another big challenge was that novel agents were administered to Western aid workers without randomisation when they were treated in Europe and the United States [5]. They proposed that instead of an RCT, suitable treatments should be trialled in parallel non-randomised studies, comparing mortality with historical data; an approach which is methodologically weaker and frequently unable to render valid evidence [5–7].

In this paper, we propose six considerations for evaluation of whether the use of placebo is ethical and illustrate their application in a primaquine antirelapse trial for *Plasmodium vivax* malaria. These considerations evolved from published literature and our experience developing, debating and fine-tuning the study design including having lengthy discussions with ethics committees. The trial, known as the Improving the Radical Cure of Vivax Malaria (IMPROV) study, has been approved by relevant ethics committees in the United Kingdom, Australia and each of the countries where the trial is underway. This double-blinded, randomised non-inferiority trial is currently recruiting patients with *P. vivax* in Indonesia, Vietnam, Afghanistan and Ethiopia [8]. All patients are treated with either chloroquine or an artemisinin combination therapy (ACT) for the acute blood stages of the infection according to national guidelines. Patients are randomised to three arms for antirelapse treatment (termed radical cure): 7 days 1 mg/kg/day primaquine (total 7 mg/kg), 14 days 0.5 mg/kg/day primaquine (total also 7 mg/kg), or 14 days placebo. The primary objective of the IMPROV study is to determine whether a 7-day high dose primaquine regimen is as safe as and non-inferior to a WHO-recommended 14-day regimen in preventing *P. vivax* relapse. The hypothesis is that a 7-day primaquine regimen is well-tolerated and non inferior to a 14-day primaquine regimen.

## Considerations for evaluation of the ethics of placebo use

The following paragraphs address our proposed considerations which are: social value; need for a randomised controlled trial and placebo; standards of care; risks of harm due to administration of placebo and harm benefit balance; clinical equipoise; and double standards.

### Social value

For any clinical study to be ethically permissible, regardless of whether it is an RCT or if placebo is employed, it must have social value relating to the importance of the information that the study is likely to produce [1]. For the IMPROV study, this is directly related to the disease burden of *P. vivax* and the knowledge gap in the treatment of the disease. *P. vivax* is a major cause of morbidity and mortality in endemic countries [9], and was estimated to be responsible for more than 14 million clinical cases globally in 2015 [10]. Much of its morbidity and mortality is attributable to the chronic relapsing nature of the infection arising from reactivation of the dormant liver stages (hypnozoites) of the parasite [11]. Preventing relapse is therefore critical to achieving malaria control and its ultimate elimination. The radical cure of vivax malaria requires treatment of both the blood stages of the parasites as well as the liver stages. The treatment of the blood stage parasites can be achieved safely and effectively with chloroquine or an ACT, however the clearance of the hypnozoites requires a hypnozoitocidal agent, primaquine, which has variable efficacy and potential for serious haemolytic toxicity. Primaquine is the only currently available hypnozoitocidal agent.

### Need for a randomised controlled trial and a placebo arm

The next question that needs to be addressed is whether there is need for a randomised controlled trial and whether an active control arm or placebo is more suitable. Without a placebo arm, it is not possible to determine if any of the treatments are effective. Although primaquine has been used clinically for more than fifty years, there are enduring questions regarding its safety and efficacy. Patients remaining in an endemic environment are vulnerable to recurrent infections due to recrudescence from the same partially treated blood stage parasite, reinfection with a new strain, or relapse from a dormant hypnozoite. The frequency and timing of relapses varies considerably with geographical location, from weeks in sub-equatorial regions to more than a year in more temperate climates [12]. Hence a control arm is vital to quantify the background risk of recurrence in the absence of treatment. Without a placebo comparator arm, equivalence between the primaquine regimens could not be used to infer that both regimens were equally efficacious, since they could both be equally useless. Furthermore, since both the disease and primaquine can cause severe haemolysis, the absence of a placebo arm makes it impossible to assess the degree to which haemolysis is attributable to the disease or the drug. In view of these confounding factors, a randomised controlled trial is vital and the non-inclusion of placebo could be considered unethical due to the fallibility of the conclusion and potentially erroneous recommendation that a trial without placebo arm could produce [13].

### Standards of care

Another consideration is the standard of care in each location. The WHO recommends treatment with chloroquine or an ACT plus primaquine for 14 days [14]. In reality, primaquine is not adopted widely in endemic areas. Two countries participating in the IMPROV study (Afghanistan and Ethiopia), do not currently recommend primaquine in routine practice. In these areas the ethical justification for the use of the 14-day placebo arm is relatively straightforward. The standard of care is no primaquine. However in countries that do recommend primaquine (Indonesia and Vietnam), the justification of a placebo arm requires further consideration.

In many endemic settings, healthcare providers are reluctant to prescribe primaquine even if it is national policy due to the uncertainty of the glucose-6-phosphate-dehydrogenase (G6PD) status of their patients and the risk of causing severe iatrogenic induced haemolysis [15]. Often the appreciable risks of severe anaemia due to the failure to prevent relapsing infections are not included into the rationale or treatment algorithm. This uncertainty is compounded by uncertainty of the clinical efficacy of the 14-day primaquine regimen, since the quality of evidence for this varies in different locations [13]. Finally, even when primaquine is prescribed appropriately, patient adherence to the 14-day regimen is thought to be poor. The *actual* standard of care in most endemic areas does not include primaquine, resulting in at least two standards: no primaquine and 14 days primaquine.

### Clinical equipoise

Clinical equipoise implies there is true uncertainty within the medical community of the risks and benefits between different study arms [16]. Clinical trials have yielded conflicting results with regard to whether 7-days 1 mg/kg primaquine (total 7 mg/kg) or 14-days 0.5 mg/kg/day primaquine (total also 7 mg/kg) is superior. Some trials have shown that the 7-day (total 7 mg/kg) primaquine regimen is “highly effective” [17], or as effective as the 14-day regimen (also total 7 mg/kg) [18], so it can be argued that there is equipoise between the two primaquine arms. In addition, neither 7-days nor 14-days primaquine has been tested against placebo, further justifying the need for a placebo arm.

### Risk of harm due to administration of placebo and harm benefit balance

This section discusses the potential direct harm caused by the administration of placebo. The main harm to an individual patient in the placebo arm is relapse, the frequency of which varies markedly with geographical location. The timing of relapse also varies, ranging from three weeks to more than a year after the initial infection [12]. Unfortunately, there is no reliable data on the timing and frequency of relapse by location, which makes this particular assessment the most difficult of all the considerations we proposed. In the IMPROV study, based on discussions with local collaborators and ethics committees, the maximum number of relapses before a patient is removed from the study is capped at three or four, depending on the site, and all relapsed infections are treated immediately. However this risk of harm is no more than the risk of harm of being treated in the clinic, which, in majority of cases, is treatment without primaquine.

An alternative assessment of harm should include comparison of the potential for direct harm of being in the placebo arm with the potential for direct harm of being in the primaquine arms, of which the most important is the risk of haemolysis. The risk of haemolysis in the 7-day primaquine arm, which has a higher individual daily dose, is theoretically higher than in the 14-day arm. However the only published data on 7-day primaquine usage, where 60 mg/day was administered, showed that this dose was safe and well tolerated [17]. The IMPROV trial excludes G6PD deficient patients from randomisation, but a limited degree of haemolysis which has no clinical significance is expected to occur in all patients, even those who are G6PD normal.

In addition to the above considerations, it is important for any study to ensure that the ancillary care obligations are met and that ancillary risks are minimised. In the IMPROV study, treatment efficacy and patient safety are ensured by close monitoring over a twelve-month follow up period following a schedule of visits and corresponding clinical and laboratory examinations [8].

### Double standards

Critics of placebo controlled trials often refer to a controversial series of multinational HIV trials in Sub-Saharan Africa in the 1990s where placebo was used as control when an effective treatment regimen had already been established. This scenario is vastly different from the context of placebo use in *P. vivax* radical cure. Firstly, good quality primaquine is cheap and widely available in a generic formulation throughout the developing world. In contrast, the main reason for not using the established standard in the AZT trials was its affordability. The HIV trials were designed to translate research findings in wealthy countries into simpler and more affordable treatment regimens in poorly resourced countries. Secondly, no superior radical cure of *P. vivax* exists in wealthy countries, hence there is no double standard of care [14].

## Discussion

Based on the above considerations, the application of placebo in the IMPROV study is ethically justified on the grounds that there is a big *P. vivax* burden and a need for a placebo-controlled trial. Furthermore, the current standard of care in most endemic places does not include prescription of radical cure. There is clinical equipoise among the study arms, the risk of harm of being in the placebo arm is that arising from the risk of relapse, and there are no double standards of care between developed and developing countries.

International, regional and national ethics guidelines set out useful general conditions for use of placebo in order to prevent double standards and to guide good practice in clinical research. For example the Declaration of Helsinki and Council of International Organisations of Medical Sciences guidelines state that placebo may be used when there is “no proven intervention” [19], or “no established effective intervention” [1]. We think that the question of whether or not a placebo arm is ethically permissible for a specific study depends on the objectives, population, and contexts of the specific study at hand.

In this paper, a set of considerations were employed to judge whether the use of placebo is justified - in a specific study in a specific context. Although these considerations evolved from a specific trial, our larger objective was to provide a starting point for unpacking the specific arguments for justifying the use of placebo in clinical trials, rather than solely relying on guidelines.

This paper focuses on placebo but these considerations also apply in observational control arms where no treatment is offered. The first is regarding the disease burden, which is related to the social value of a trial [1, 20]. This is an important requirement for any clinical research, not only placebo controlled trials. The second is whether there is a need for a randomised controlled trial and if other study designs are more scientifically appropriate or more acceptable by the community. For instance in some cases, historical controls or observational studies such as those suggested for the Ebola trials could be adopted [6]. These study designs may not yield the most robust data but may be more practical and acceptable by the community in situations of epidemic and fear.

If randomised controlled trials are the right design, the next question relates to the control arm. The default should be the standard of care, about which important questions must be asked. These include the nature of the evidence for the standard of care and if the standard is reasonable in the context. Are there costs, availability, safety, geographical, genetic, or medical differences due to age, gender, pregnancy or comorbidities that render the apparent “standard of care” non-standard?

The most important condition is that patients should not be knowingly allowed to suffer from serious harm if they are in the placebo arm. This condition applies even if there is no proven treatment. For example, it has been argued that placebo should not be used if there is high mortality such as in the case of the Ebola trials [5]. This is a pre-condition to a favourable harm-benefit ratio that renders a study ethically acceptable.

While the above criteria apply to all placebo controlled trials wherever they are conducted, the issue of double standards is particular to low-income settings. As a default, double standards are not ethically permissible unless there are compelling reasons, which are specific to the trial and specific to the context. The threshold for the acceptability of double standards should be set high; double standards must not be a rule but an exception.

For any clinical trial to be ethical regardless of whether a placebo is used or not, it must first fulfil several universal requirements, for example it must be scientifically valid, be responsive to local healthcare needs, must minimise the risks to which the participants are exposed, and must not be exploitative [20]. For the purpose of focus, this paper does not address these or other equally important ethical issues specific to the trial such as the non-inferiority study design, the role of a Data and Safety Monitoring Board, procedures which are in place in case of haemolysis in the primaquine arms, the treatment regimen if patients have relapses, the treatment for G6PD deficient patients, compensation for any increased burdens of being in the trial, post-trial access, informed consent procedures, as well as the quality of the placebo and blinding. These have been partially addressed in the published protocol and are beyond the scope of this paper [8].

## Conclusions

In this paper, we present a set of considerations to systematically evaluate the ethics of placebo controlled trials. We propose that similar considerations should be prospectively applied to other placebo controlled trials and observational control arms where no treatment is offered.
